# Biosecurity and antimicrobial use in broiler farms across nine European countries: toward identifying farm-specific options for reducing antimicrobial usage

**DOI:** 10.1017/S0950268822001960

**Published:** 2022-12-27

**Authors:** Panagiotis Mallioris, Gijs Teunis, Giske Lagerweij, Philip Joosten, Jeroen Dewulf, Jaap A. Wagenaar, Arjan Stegeman, Lapo Mughini-Gras, the EFFORT consortium, H. Graveland, H. Schmitt, D. Mevius, A. van Essen, B. Gonzalez-Zorn, G. Moyano, P. Sanders, C. Chauvin, J. David, A. Battisti, A. Caprioli, T. Blaha, K. Wadepohl, M. Brandt, F. Aarestrup, T. Hald, S. Duarte, D. Wasyl, D. Krasucka, B. Biernacki, J. Szumilo, H. Daskalov, H. Saatkamp, K. Stärk

**Affiliations:** 1 Division of Infectious Diseases and Immunology, Faculty of Veterinary Medicine, Utrecht University, Utrecht, the Netherlands; 2 Institute for Risk Assessment Sciences, Utrecht University, Utrecht, the Netherlands; 3 National Institute for Public Health and the Environment, Centre for Infectious Disease Control, Bilthoven, the Netherlands; 4 Veterinary Epidemiology Unit, Department of Internal Medicine, Reproduction and Population Medicine, Faculty of Veterinary Medicine, Ghent University, Ghent, Belgium; 5 Division of Farm Animal Health, Faculty of Veterinary Medicine, Utrecht University, Utrecht, the Netherlands

**Keywords:** Antibiotics, antimicrobial use, random forest, mixed-effects random forest, broilers, biosecurity

## Abstract

Broiler chickens are among the main livestock sectors worldwide. With individual treatments being inapplicable, contrary to many other animal species, the need for antimicrobial use (AMU) is relatively high. AMU in animals is known to drive the emergence and spread of antimicrobial resistance (AMR). High farm biosecurity is a cornerstone for animal health and welfare, as well as food safety, as it protects animals from the introduction and spread of pathogens and therefore the need for AMU. The goal of this study was to identify the main biosecurity practices associated with AMU in broiler farms and to develop a statistical model that produces customised recommendations as to which biosecurity measures could be implemented on a farm to reduce its AMU, including a cost-effectiveness analysis of the recommended measures. AMU and biosecurity data were obtained cross-sectionally in 2014 from 181 broiler farms across nine European countries (Belgium, Bulgaria, Denmark, France, Germany, Italy, the Netherlands, Poland and Spain). Using mixed-effects random forest analysis (Mix-RF), recursive feature elimination was implemented to determine the biosecurity measures that best predicted AMU at the farm level. Subsequently, an algorithm was developed to generate AMU reduction scenarios based on the implementation of these measures. In the final Mix-RF model, 21 factors were present: 10 about internal biosecurity, 8 about external biosecurity and 3 about farm size and productivity, with the latter showing the largest (Gini) importance. Other AMU predictors, in order of importance, were the number of depopulation steps, compliance with a vaccination protocol for non-officially controlled diseases, and requiring visitors to check in before entering the farm. *K*-means clustering on the proximity matrix of the final Mix-RF model revealed that several measures interacted with each other, indicating that high AMU levels can arise for various reasons depending on the situation. The algorithm utilised the AMU predictive power of biosecurity measures while accounting also for their interactions, representing a first step toward aiding the decision-making process of veterinarians and farmers who are in need of implementing on-farm biosecurity measures to reduce their AMU.

## Introduction

Antimicrobial use (AMU) is known to drive the emergence and spread of antimicrobial resistance (AMR) at the human-animal-environment interface [1]. However, therapeutic AMU in livestock is an essential component of animal health and welfare [2]. Regrettably, in several parts of the world, antimicrobials are still used as growth promoters [3]. Prudent use of antimicrobials is essential for optimising their efficiency by minimising the associated risks of AMR. As AMU in animals also leads to contamination of animal-derived products with drug residues, AMU risks do not only include antimicrobial resistant infections *per se*, but also various negative effects for human health and the environment [4].

Previous studies have estimated that 73% of all antimicrobials sold globally are used in livestock farming [5]. In 2017, global consumption of all antimicrobials in food-producing animals (chicken, cattle and pigs) has been estimated at 93 309 tons and has been projected to reach 104 079 tons by 2030 if no measures are taken [6], with a considerable portion of this increase being attributed to poultry [7]. Specifically, for the same year it has been estimated that chickens consumed about 68 mg/PCU (population correction units) of antimicrobials, on average, and that the sector will contribute to 33% of the estimated global increase in AMU by 2030 [6]. This is because worldwide, and especially in low- and middle-income countries, poultry is one of the most rapidly growing farm animal sector and is expected to expand even further as poultry farming continues to be intensified, a practice that is closely linked to the need for AMU [8–10].

Due to these concerns, there is a growing interest in promoting farm-level practices in the poultry sector that minimise (the need for) AMU and consequently, (the emergence/spread of) AMR and the likelihood of antimicrobial residues contaminating the food chain as well as the environment. Key components of such practices are internal and external farm biosecurity, vaccination, water and feed quality, special attention to day-old chick development and micro-climate conditions within the barn, among others [11, 12]. While information on these key components is available in the literature, very few studies exist that have assessed the association of these practices with AMU in poultry farming and even less in broilers specifically [13–15]. Due to considerable variation in AMU in poultry flocks, both within and between countries [16], the effects of some of those practices vary substantially from one farm to another. Therefore, confusion and uncertainty may arise when recommendations are to be given on which farm-level interventions would be most suitable to reduce AMU in a given situation. This variation has also been observed in studies regarding specific on-farm practices and introduction or spread of zoonotic pathogens. For example, varying hygiene procedures of the catching crews influence the risk of colonisation with *Campylobacter* when partial depopulation is applied [17]. Similarly, disinfection baths have also been found to be inadequate biosecurity measure unless paired with appropriate hygienic practices, such as scrubbing visible manure before soaking in a clean disinfection bath for the by-the-manufacturer-recommended time [18]. These observations not only show the importance of biosecurity in reducing disease burden, but also the significance of accounting for farm-specific settings, as the interaction between varying biosecurity practices determines whether an individual measure will be effective or not. Although little research exists on the relationship between specific biosecurity measures and AMU, previous studies on groups of measures, or internal and external biosecurity more generally, have shown that they can reduce AMU on broiler farms [19, 20]. The implementation of farm-specific interventions in these studies indicates that quantifying the effects of different AMU reduction options for a given farm is important to curtail AMU in a feasible and pragmatic manner. These effects, along with their economic impact assessment, provide a foundation for decision making on the development of customised AMU reduction plans.

The aim of this study was to identify and quantify the main biosecurity measures (as defined by the Biocheck.UGent™ broiler survey) associated with AMU in broiler farms across nine European countries. Moreover, based on these biosecurity measures, we aimed at developing an algorithm that generates farm-specific AMU reduction plans, which include the best set of biosecurity measures (in terms of AMU reduction) to implement on the farm and their respective estimated costs. The analysis was therefore composed of three parts: (i) a risk factor analysis of biosecurity measures associated with AMU in broiler farms, (ii) the development of a scenario-based algorithm that identifies the best interventions for a given farm and quantifies their effect on AMU reduction, (iii) a cost-effectiveness analysis of the proposed biosecurity measures. This algorithm is a first step toward the development of an assistive advising tool for veterinarians and/or farmers and possibly also regulatory bodies when plans need to be made to reduce AMU at the farm-level.

## Materials and methods

### Data collection

This study used data collected within the EFFORT project (Ecology from Farm to Fork Of microbial drug Resistance and Transmission; http://www.effort-against-amr.eu/). For a detailed description of the study design, including farms selected, data collection methods and the specific information collected, we refer to [21]. Briefly, in May–June 2014, AMU data were collected at 181 farms located in nine European countries (Belgium, Bulgaria, Denmark, France, Germany, Italy, the Netherlands, Poland and Spain); 20 farms were sampled in each country (except in Belgium; *n* = 21), using a cross-sectional study design. Eligibility criteria for farms were defined in agreement with local farming organisation and convenience (e.g., distance). Consequently, the sample of farms in each country cannot be considered representative for the broiler production in that specific country, but altogether these farms provide a snapshot of the situation across the nine countries in question. Biosecurity data for the farms were collected using the Biocheck.UGent™ survey for broilers [15]. Additionally, AMU was recorded as treatment incidence (TI) based on Defined Daily Dose (DDDvet) per 1000 animal-days at risk. TIDDDvet can be read as the percentage of time that a broiler is treated with antimicrobials in its life. For example, when TIDDDvet on a farm equals 150, the broilers on that farm were treated during 15% (i.e. 150/10) of their life. The method used for the quantification of AMU is described in detail by [16]. In this study, the relationship between the biosecurity related factors with the total sum of TIDDDvet on farm (based on sales data) was assessed. For the analysis, TIDDDvet values were transformed using Equation (1) [22] to deal with the presence of a handful outliers, as it avoids zero values becoming infinite in contrast to the standard natural logarithm transformation.1
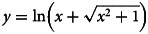


The number of workers and average number of broilers per round were transformed using the natural logarithm due to a few outliers in the former and for rescaling in the latter. The biosecurity related factors with more than 10% missing values or less than 15% variation were excluded from all analyses, as done before by [23]. For the rest of the missing data the missing indicator method was used. Table S1 shows the descriptive statistics of these factors. All farms were anonymised to ensure that results could not be traced back to individual farms. Country was also anonymised, as this was required by the farming organisations, as done in other studies based on the EFFORT data [16, 21, 24] .

### Risk factor analysis

**Fig. 1.**
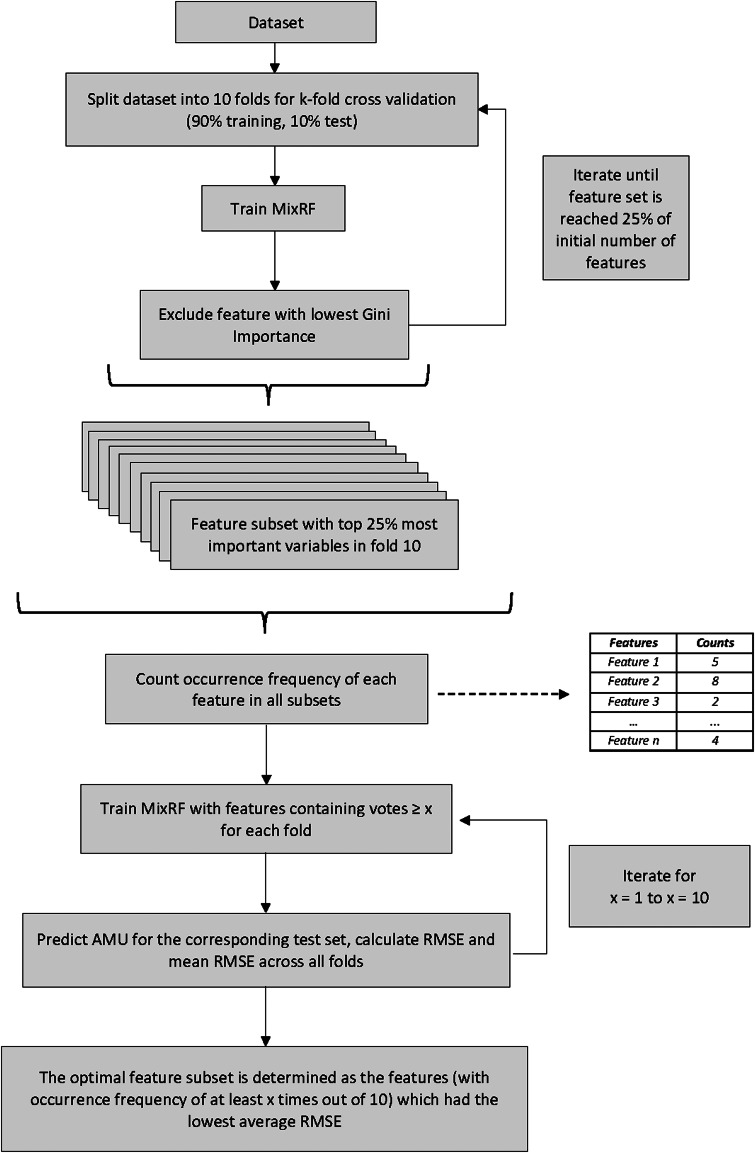
Steps of the Random Forest Recursive Feature Elimination (RF-RFE) procedure.

A Random Forest (RF) analysis was conducted to identify risk factors for AMU. Specifically, given the clustered nature of the data, a mixed-effects RF model [25] was used, i.e. mixed-effects random forest (Mix-RF) [26], with a random intercept at the country level. An advantage of RF is its ability to capture interaction effects without the need of defining them [27]. Variable selection was implemented with the Recursive Feature Elimination algorithm for automatic determination of optimal feature subsets in the RF setting (RF-RFE) [28]. The RF-RFE algorithm allows for an automated selection of feature subsets that is optimised for prediction based on Gini Importance and a goodness-of-fit metric, in this case the Root Mean Square Error (RMSE). The exact steps of the RF-RFE are visualised in Figure 1. Briefly, a ten-fold cross validation (ten-fold CV) was initially applied to create the train and test sets (90% and 10% of observations respectively). Starting by fitting all variables, a Mix-RF model was then run iteratively with the least important feature, based on Gini Importance, being removed in each iteration until the top 25% of features in terms of importance remained (i.e. top 13 features out of 53). Next, across all ten importance-optimised subsets, the feature frequencies were calculated and based on these frequencies the predictors were grouped together, i.e., predictors appearing at least once in the ten folds, at least twice, at least three times, etc., until ten out of ten (i.e., the maximum number of appearances, meaning that the feature was present in all folds). For each of these frequency-based subsets, the RMSE across all ten test sets using each respective train set from the initial ten-fold CV was estimated. Finally, the subset with the lowest average RMSE represented the final multivariable Mix-RF model. At this point, the Mix-RF model was also tuned with regard to the number of trees to grow and number of predictors selected in each split and the default values (i.e. number of trees = 500 and *m* = total number of predictors/3 = 21/3 = 7) performed the best.

**Fig. 2.**
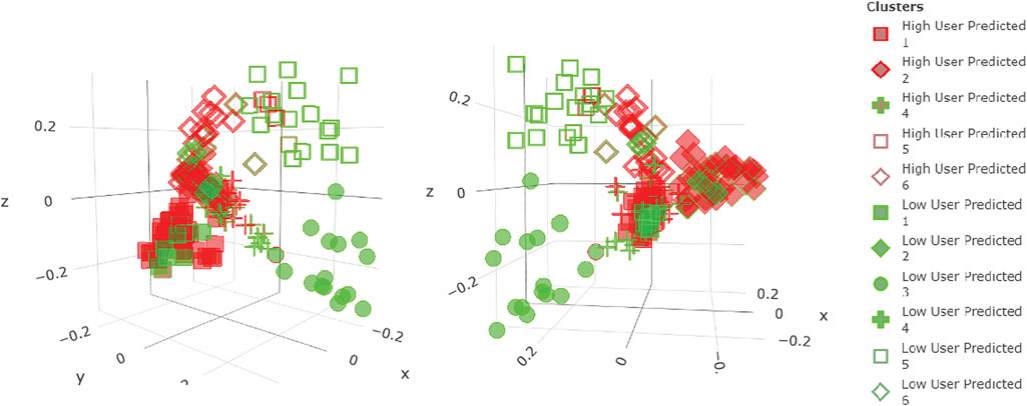
3D MDS plot of Random Forest's proximity matrix; each dot represents a farm and the distance between the dots represents dissimilarity (the further away the more different). The shape of the dots represents the different clusters, the inner colour displays the real AMU class and the outer stroke colour represents the predicted AMU class (in both cases red represents High user and green Low user; if the image is shown in B&W, red is more intense.

Using the final multivariable Mix-RF model with the whole data set, the similarities among farms were quantified with their proximity matrix. The proximity matrix is constructed by comparing how often two observations end up in the same terminal node across all trees within the RF. This is then divided by the number of trees (500 in this analysis) to normalise the proximity score between 0 and 1, where 0 denoted complete dissimilarity and 1 complete similarity with respect to the RF prediction [29]. This matrix after subtracted from 1 to reflect dissimilarities was then projected onto a plot using multidimensional scaling (MDS) [30] using the R package ‘randomForest’ [31]. For the purpose of this projection, the response value was also considered for visualisation. The real and predicted transformed TIDDDvet values were normalised between 0 and 1, with those larger than 0.5 being denoted as ‘High User’ of antimicrobials and smaller than 0.5 as ‘Low User’. This three-dimensional graph can be seen in Figure 2.

From the MDS plot, groups of similar farms were identified using k-means clustering [32] with the R package ‘mclust’ [33]. The optimal number of clusters was determined using the Bayesian information criterion (BIC) [34]. In each cluster, the prototypes for each class were computed (i.e. the representative value of a predictor for a class in a cluster; in our cases the classes were those described above i.e. the High and Low Users predicted). Usually, these are the values of a class' average observation in a cluster, i.e., the observation that within each cluster has the largest sum of proximity scores with the rest of the observations in its class. To aid interpretation, the prototypes here were calculated by taking for all observations in a cluster's class the median for numerical features and the highest frequency value for categorical features. The purpose of this cluster analysis was to determine statistically whether and which farms were more alike than others and to use the prototypes as a main descriptive measure of the patterns observed between Low and High Users in each cluster.

### Algorithm for farm-specific AMU reduction plans

**Fig. 3.**
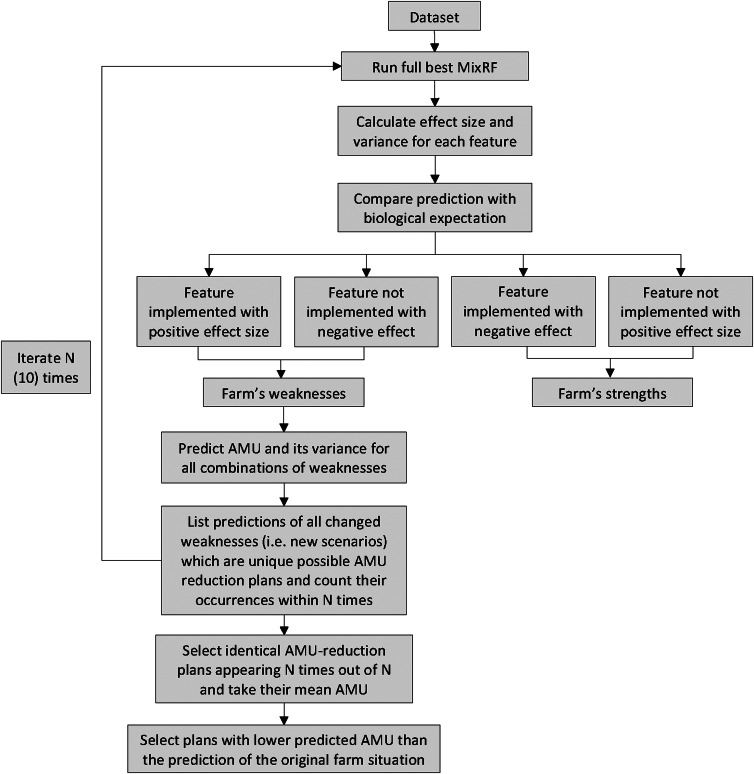
Visualisation of the steps to formulate AMU-reducing plans for a farm.

Based on the final multivariable Mix-RF model built for the risk factor analysis, an algorithm was developed for generating customised AMU-reducing plans for a given farm. This farm could be either part of the original dataset or completely new. The algorithm identified possible solution plans and assessed their efficiency and feasibility for a particular situation in a quantifiable and systematic manner. The structure and steps of the algorithm are visualised in Figure 3 and are as follows:

- The initial step consisted of extracting the effect sizes of each predictor within the final Mix-RF model. That was done based on the method of partial dependence using the R package ‘rfUtilities’ [35, 36]. As this package requires predictors to be numerical, our categorical predictors (all were binary with a few having a third category for missing values) were reclassified as such and the values for the missing category were imputed using Multivariate Imputation by Chained Equations ‘mice’ package in R with RF [37]. For each effect size, a bootstrapped 95% confidence interval (CI) was calculated using the R package ‘randomForestCI’ (with 1000 iterations) [38]. The estimated effect sizes of each feature can be interpreted as the effect on the response (i.e. TIDDDvet) for a one unit change in the predictor, while averaging over the effects of all other variables [36].
- Since all of our candidate predictors were binary, this information placed in a cross table was used to classify each feature as a farm's strength or weakness in terms of AMU. As shown in Figure 3, strengths were defined as the variables that were implemented by the farm and had a negative effect size or were not implemented and had a positive effect size on AMU. Similarly, weaknesses were defined as those features that were not implemented and had a negative effect size or were implemented and had a positive effect size. The variables marked as farm's weaknesses represented the action points at the farm-level.
- Because these features were assessed only based on their AMU prediction ability, their connotation had also to be assessed biologically for valid inference. Thus, researchers' knowledge was incorporated by assessing the direction of the association (positive or negative) as compared to the expected biological interpretation of the biosecurity measure (Table 2). If there was a match, then the variable was selected for inclusion in the solution plan.
- After selection of all candidate interventions, a scenario-based analysis was implemented based on all possible combinations of these variables. This means that a unique scenario was created for each possible combination of variables, the final Mix-RF model was used to predict each respective AMU and the scenarios with lower predicted AMU than the farm's original situation prediction were selected.

After several runs, it was noted that some predictors with low Gini Importance could have effect sizes with opposite signs (associations) in the different runs of the Mix-RF model due to the uncertainty intrinsic in the RF estimations. To address this, the previous steps (i, ii, iii and iv) were applied iteratively ten times and the scenarios identified by the algorithm included only those variables with 10/10 consistency in association; the corresponding mean AMU value across the iterations was then reported. The aforementioned steps were structured in such a way that they filtered out systematically variables that showed evidence of non-causative relationships with AMU. Nevertheless, this does not necessarily establish a causative relationship of the unfiltered ones, just lack of the former.

### Cost-effectiveness analysis of AMU reduction plans

The final output of the aforementioned algorithm provided scenarios that reflected a particular farm of interest applying a specific set of biosecurity measures and predicting its respective AMU. The final part in identifying the most suitable intervention scenarios for that farm was the assessment of its economic impact. The cost-effectiveness analysis here aimed at calculating the additional costs introduced due to the interventions relative to the AMU reduction expected to be achieved. The respective costs of the interventions were quantified by taking into account both the implementation and operational costs. The implementation costs were the purchasing costs of the relevant equipment along with depreciation and its installation costs, if any. The operational costs reflected the maintenance costs of the respective interventions. Their profile was measure-specific, but overall it concerned material and utility costs, as for example the cost of disinfectant that needs to be refilled, or the purchasing of plates for hygienograms, among others. These costs could also be dependent on production characteristics, such as the number of animals and number of rounds per year, e.g. vaccination costs variate according to these factors. All expenses were determined on a yearly basis for all features present in the final multivariable Mix-RF model and are displayed on Table 1. These costs were not country-specific, but based on indicative prices in the Netherlands and Belgium (the prices can also be redefined manually within the algorithm). The cost was also expressed per unit of TIDDDvet by dividing the total cost of the scenario with the difference of scenario's TIDDDvet and original TIDDDvet prediction. All analyses were performed in the open-source environment R version 4.0.3 [39].

**Table 1.** Costs of biosecurity measures for the solution plan algorithm

Biosecurity measures	Implementation cost €	Operational cost €/y	Years to last	Depreciation cost^a^ €/y	Total cost €/y	References assumptions and comments
bqq023_Visitors/traders are not allowed to enter the stables/have direct contact	–	–	Inf.	–	0	No costs
bqq024_The poultry house is depopulated in one step ^b^	Farm size/10	–	Inf.	–	Farm size/10	Indicative cost and arbitrarily was set to 1/10^th^ less broilers are produced × 2 kg of meat × 1 €/kg
bqq033_When checking water quality, the water sample is taken at the nipple	–	–	Inf.	–	0	No costs
bqq045_Visitors are obliged to check in before having entrance to the stables	–	–	Inf.	–	0	No costs
bqq050_Visitors/personnel are obliged to wash/disinfect hands before entering	72 + 35.9	–	10	7.2	115.1	A dispenser costs 72€ www.agrologic.be, assume 10 years amortisation following a linear depreciation and no salvage price. A refill of a hand soap costs 3.59 www.agrologic.be. Assume 10 refills per year. [53]
bqq055_Specific preventive measures are taken for the material supply	72 + 71.8	–	10	7.2	151	Cost of disinfectant [53]
bqq060_The farm is fenced off ^b^	5000 × number of stables	–	25	(5000 × number of stables)/25	26/25 × 5000 × number of stables	Indicative cost
bqq066_There is natural stagnant water or running water within less than 1 km of the farm	–	–	–	–	–	Not available
bqq075_There is a farm specific protocol for vaccination of non-officially controlled diseases that is complied with	0.02399 × Farm size × Production rounds	–	–	–	0.02399 × Farm size × Production rounds	Cost of a vaccine × Farm size × Production rounds https://veteriankey.com/poultry-vaccines/
bqq078_The poultry density of the poultry house ≥ 38 kg/m^2^ ^b^	Farm size/10	–	–	–	Farm size/10	Indicative cost and arbitrarily was set to 1/10^th^ less broilers are produced
bqq080_There are disinfection baths for vehicles present at the entry of the company	5200	–	–	–	5200	20 € × 5 L of disinfectant per week × 52 weeks in a year https://www.sunstar-shop.nl
bqq084_The efficacy of cleaning/disinfection is checked by taking a hygienogram	80	4 × 10	–	–	120	This is considered with the logic that taking a hygienogram has the goal to reduce its result. Price of taking hygienogram 4 times per year and a labour cost of 10 euro/h [53]
bqq087_There is a FARM-hygiene lock present ^b^	6000	–	10	–	6000	Indicative price
bqq096_The drinking water system is fully disconnected and cleaned/disinfected	20 × 10	10 × 8	Inf.	–	280	One full day of labour (8 h × 10 Euro) + cost of disinfectant 10L.
bqq101_Material is stored per stable recognisably	130 × number of stables	–	5	–	130 × number of stables	130 is the cost of several items per stable [53]
bqq102_Stable specific clothing is available	(15 + 30) × (number of workers + 5) × number of stables	–	3	–	(15 + 30) × (number of workers + 5) × number of stables	45 is indicative cost for overall clothing and boots for workers and visitors [53]
bqq103b_Roof ventilation is used in the poultry house	–	–	–	–	–	Not included as a proposed intervention
bqq103e_Length ventilation is used in the poultry house	–	–	–	–	–	Not included as a proposed intervention

a Depreciation cost is implementation cost divided by years to last.

b The costs of these variables were arbitrarily set due to lack of information and/or assumptions for properly calculating them as they can variate extremely depending on farmers decisions and opportunities.

## Results

### Risk factors

The lowest RMSE within the RF-RFE algorithm was obtained with features appearing at least six times out of ten and this gave a model of 21 variables with an RMSE of 1.58 on the transformed scale. Based on Gini Importance, the top three features where: (i) the number of broilers per round, (ii) the number of production rounds per year and (iii) the number of workers, which are all proxies for farm size and productivity and are thus considered as the ‘null model’ (i.e. the model with all necessary *a priori* control covariates). All variables in the null model had negative effect sizes (i.e. AMU is reduced as they increase). The other variables in the model were ten for internal biosecurity and another eight for external biosecurity. The heuristic theoretical importance limit of 1/N (with N being the number of factors in the model) was used to identify which variables to describe here as most important (i.e., variables which percentage contributions to the total Gini Importance is bigger than 1/21 × 100% = 4.7%). After the null model, this limit included also the following variables: ‘visitors are obliged to check in before entering the stables (reference: no *vs.* yes)’, ‘a specific protocol for non-officially controlled diseases is applied on farm (reference: no *vs.* yes)’ and ‘in how many steps the poultry house is depopulated (reference: one *vs.* two or more)’. These three variables had a positive association with AMU. The full model with count frequency, Gini Importance, effect sizes with their bootstrapped 95% CI and biosecurity block for each variable is shown in Table 2.

**Table 2.** Optimal feature subset in final multivariable mixed-effects random forest model obtained through the Recursive Feature Elimination algorithm with antimicrobial usage in TIDDDvet as outcome

Features	Count	Gini importance index	Effect size (95% CI)	Sub-block (Biological expectation)
Average number of chicks per round (null model; ln transformed)		93.0	−0.046 (−0.053 to −0.038)	Null model (+)
Average number of rounds per year (null model)		77.1	−0.126 (−0.132 to −0.12)	Null model (+)
Number of people working at the poultry farm in total (null model; ln transformed)		72.4	**−0.118** (**−0.128** to **−0.108)**	**Null model** (**−)**
Are visitors obliged to check in before having entrance to the stables? 0 – no 1 – yes	9	37.2	0.488 (0.458–0.518)	External (**−**)
In how many steps is the poultry house depopulated? 0 – In one step 1 – In two or more	7	28.9	**0.307** (**0.294–0.32)**	**External** (**+)**
Is there a farm specific protocol of non-officially controlled disease that is complied with? 0 – no 1 – yes	6	28.7	0.51 (0.469–0.55)	Internal (**−**)
Is drinking water system fully disconnected and cleaned/disinfected? 0 – not after every round 1 – after every round	9	22.2	**−0.519** (**−0.547** to **−0.49)**	**Internal** (**−)**
Are there disinfection baths for vehicles present at the entry of the company? 0 – No 1 – Yes	7	20.3	0.225 (0.21–0.24)	Internal (**−**)
Length ventilation is used in the sampled barn 0 – No 1 – Yes	7	19.8	0.318 (0.304–0.332)	External (NA)
What is the poultry density in the sampled house? 0 – ≤ 37 kg/m^2^ 1 – ≥ 38 kg/m^2^	7	19.5	−0.048 (−0.058 to −0.038)	Internal (+)
Is the farm fenced-off? 0 – not completely 1 – completely	7	18.6	0.157 (0.149–0.166)	External (**−**)
Are specific preventative measures taken for material supply? 0 – no 1 – yes	8	15.9	**−0.205** (**−0.216** to **−0.195)**	**External** (**−)**
Is stable-specific clothing available? 0 – no 1 – yes	7	14.1	**−0.109** (**−0.115** to **−0.103)**	**Internal** (**−)**
Is efficacy of cleaning/disinfection checked by taking a hygienogram? 0 – never 1 – not never	6	13.4	0.068 (0.062–0.075)	Internal (**−**)
Is material stored per stable and recognisable? 0 – no 1 – yes	7	12.7	**−0.058** (**−0.065** to **−0.051)**	**Internal** (**−)**
Are visitors/personnel obliged to wash/disinfect hands before entering? 0 – no 1 – yes	6	12.5	0.141 (0.134–0.148)	External (**−**)
Is there a FARM-hygiene lock present? 0 – No 1 – Yes	6	12.1	**−0.071** (**−0.078** to **−0.065)**	**Internal** (**−)**
Allowed for visitors/traders to enter stables/have direct contact? 0 – never 1 – not never	6	11.8	−0.091 (−0.1 to −0.082)	External (+)
When checking water quality, where is the water sample taken? 0 – not at nipple 1 – at nipple	7	11.5	**−0.095** (**−0.101** to **−0.088)**	**External** (**−)**
Roof ventilation is used in the sampled barn 0 – No 1 – Yes	7	11.0	−0.101 (−0.107 to −0.096)	External (NA)
Is there natural stagnant or running water within less than 1 km of farm? 0 – no 1 – yes	6	10.6	−0.006 (−0.011 to −0.002)	External (+)

The features are ordered by Gini Importance index. In the fourth and fifth columns in bold are the variables for which their effect size sign and biological expectation of the measure match and thus they satisfy one of the criteria for being selected as candidate intervention on the farm.

### Clustering and prototypes

Based on the BIC, six clusters were identified as optimal for the k-means clustering on the proximity matrix of the final multivariable Mix-RF model (Fig. 2). Figure S1 shows the number of farms per country included in each cluster. All but Cluster 3 (Low Users only) contained both Low and High Users thus, in that cluster, comparison of the prototypes between High and Low Users was not applicable. The prototypes for all clusters, as well as for all farms altogether, can be viewed in Table 3. Regarding the null model variables, at the overall level (i.e. all farms as one cluster), the median number of chickens per round was lower for Low Users (34 550 *vs.* 43 000), but at the cluster level for 3 out of 6 clusters that was not the case (Cluster 2: 100 400 *vs.* 80 000; Cluster 4: 83 500 *vs.* 34 925; and Cluster 6: 30 500 *vs.* 26 000). The average number of rounds had a range of 5 to 8 per year with the Low Users having more rounds, both overall and at the cluster level. Cluster 3, which had only Low Users, had eight rounds, as also the Low Users of Cluster 4. Regarding the number of workers, overall and in Cluster 5 Low and High Users had around two workers. In four of the other clusters, High Users had also a median of 2 workers and Cluster 3 had 1. Apart from Cluster 2 (where Low Users had 1.5 as median) Low Users had more workers than their respective High User class with maximum difference in Cluster 1 (median of 7.5 workers for Low Users). With regard to being obliged to check in before being granted entrance to the stables, overall that measure was applied by the majority of both Low and High Users (52% and 97% respectively). In Clusters 1, 2, 3, 4 and 6, both classes applied the measure, and in Cluster 5 100% of either did not. Vaccination protocol for non-officially controlled diseases was applied by the majority of farms in all clusters and overall, with the exception of 100% of Low Users in Cluster 3. Interestingly in Cluster 1, 83% of Low Users and 94% of High Users applied the measure while for Cluster 5, those numbers were 94% *vs.* 67% respectively. Finally, the depopulation steps equalled one for most of Lower Users, overall and in Clusters 3, 4 and 5. In Cluster 4, the majority of High Users had also one depopulation step, while in Cluster 5 High Users applied two or more depopulation steps. In Clusters 1, 2 and 6, both High and Low Users mostly applied two or more depopulation steps.

**Table 3.** Prototypes of clusters identified in the proximity matrix of the final multivariable mixed-effects random forest model; a prototype is the representative value of class in a cluster; for numerical values that is the median and for binary the highest frequencies of 0 or 1

Feature	Gini Importance in %	Predicted Usage	Cluster 1		Cluster 2		Cluster 3		Cluster 4		Cluster 5		Cluster 6		All farms as one cluster	
			value	%	value	%	value	%	value	%	value	%	value	%	value	%
TIDDDvet _What is your current antimicrobial usage?		**Low User**	0		4.77		0		7.34		8.9		8.9		4.37	
		**High User**	339.48		91.71		–		85.73		102.9		144.2		146.23	
Predicted TIDDDvet		**Low User**	9.37		13.39		0.72		10.74		10.3		23		5.69	
		**High User**	267.13		92.9		–		90.57		56.91		128.51		141.09	
bqq011_How many chickens are there for each set-up/round on average?	16.2%	**Low User**	18 000		100 400		34 800		83 500		30 000		30 500		34 550	
		**High User**	40 000		80 000		–		34 925		44 880		26 000		43 000	
bqq002_How many people are working at the poultry farm in total?	13.5%	**Low User**	7.5		1.5		1		2.5		2		3		2	
		**High User**	2		2		–		2		2		2		2	
bqq010_How many rounds do you have on average?	12.9%	**Low User**	5.75		7.5		8		8.2		6		6.5		7.45	
		**High User**	5.5		7		–		7.5		5		5.5		6	
bqq045_Are visitors obliged to check in before having entrance to the stables?	6.9%	**Low User**	1	100%	1	100%	1	63%	1	100%	0	100%	1	67%	1	52%
		**High User**	1	100%	1	100%	–		1	100%	0	100%	1	95%	1	97%
bqq075_Is there a farm specific protocol for vaccination of non-officially controlled diseases that is complied with?	5.2%	**Low User**	1	83%	1	100%	0	100%	1	67%	1	94%	1	100%	1	60%
		**High User**	1	94%	1	100%	–		1	72%	1	67%	1	100%	1	93%
bqq024_In how many steps is the poultry house depopulated?	5.1%	**Low User**	1	83%	1	100%	0	94%	0	67%	0	65%	1	100%	0	62%
		**High User**	1	66%	1	82%	–		0	67%	1	100%	1	95%	1	72%
bqq096_Is the drinking water system fully disconnected and cleaned/disinfected?	3.9%	**Low User**	0	67%	1	100%	0	81%	0	50%	0	71%	0	100%	0	70%
		**High User**	0	98%	1	100%	–		1	56%	0	100%	0	100%	0	62%
bqq080_Are there disinfection baths for vehicles present at the entry of the company?	3.6%	**Low User**	1	100%	0	100%	0	100%	0	83%	0	100%	0	100%	0	86%
		**High User**	1	96%	0	100%	–		0	100%	0	100%	0	95%	0	63%
bqq103e_Is length ventilation used in the poultry house?	3.5%	**Low User**	0	100%	0	50%	0	88%	0	67%	0	94%	0	100%	0	88%
		**High User**	0	76%	1	77%	–		0	50%	0	67%	0	86%	0	58%
bqq060_Is the farm fenced off?	3.4%	**Low User**	1	83%	0	100%	0	100%	0	83%	0	71%	0	67%	0	76%
		**High User**	1	58%	0	74%	–		1	56%	0	67%	1	67%	0	51%
bqq078_What is the poultry density of the poultry house?	3.3%	**Low User**	0	50%	1	100%	1	88%	1	100%	1	88%	1	67%	1	84%
		**High User**	0	74%	1	97%	–		1	61%	1	100%	1	62%	1	60%
bqq055_Are specific preventive measures taken for the material supply?	2.6%	**Low User**	1	83%	0	100%	1	56%	0	67%	0	71%	0	100%	0	58%
		**High User**	0	54%	0	97%	–		0	89%	0	100%	0	86%	0	78%
bqq084_Is the efficacy of cleaning/disinfection checked by taking a hygienogram?	2.6%	**Low User**	1	67%	1	100%	0	50%	1	100%	0	100%	0	67%	0	58%
		**High User**	1	64%	1	97%	–		1	78%	0	67%	0	67%	1	70%
bqq102_Is stable specific clothing available?	2.5%	**Low User**	1	83%	0	100%	1	63%	1	67%	1	53%	1	100%	1	62%
		**High User**	1	52%	0	56%	–		0	56%	0	67%	0	62%	0	54%
bqq101_Is material stored per stable recognisably?	2.5%	**Low User**	1	100%	0	100%	1	75%	1	83%	0	53%	1	67%	1	66%
		**High User**	1	62%	1	59%	–		1	67%	0	67%	1	52%	1	60%
bqq087_Is there a FARM-hygiene lock present?	2.4%	**Low User**	1	100%	1	100%	1	100%	1	67%	1	71%	1	100%	1	86%
		**High User**	1	78%	0	77%	–		0	78%	1	67%	1	57%	1	50%
bqq050_Are visitors/personnel obliged to wash/disinfect hands before entering?	2.1%	**Low User**	1	100%	0	50%	1	94%	0	50%	0	94%	1	67%	1	56%
		**High User**	1	90%	0	87%	–		0	78%	0	100%	0	62%	0	53%
bqq033_When checking water quality, where is the water sample taken?	2.1%	**Low User**	0	67%	1	100%	0	50%	1	67%	1	59%	1	67%	1	56%
		**High User**	0	54%	1	67%	–		1	72%	1	100%	1	71%	1	61%
bqq023_Are visitors/traders allowed to enter the stables/have direct contact?	2.1%	**Low User**	0	100%	1	100%	0	69%	0	83%	1	100%	1	67%	1	54%
		**High User**	0	92%	0	80%	–		0	89%	1	100%	1	81%	0	74%
bqq103b_Is roof ventilation used in the poultry house?	1.9%	**Low User**	1	67%	1	100%	0	69%	1	67%	0	100%	0	67%	0	68%
		**High User**	0	68%	0	54%	–		0	56%	0	100%	0	95%	0	67%
bqq066_Is there natural stagnant water or running water within less than 1 km of the farm?	1.6%	**Low User**	0	100%	0	50%	1	88%	0	50%	1	59%	0	100%	1	56%
		**High User**	0	52%	1	69%	–		1	61%	1	67%	0	57%	1	56%

### Cost-effectiveness analysis of farm-specific AMU reduction plans

As an example to illustrate the outcome of the cost-effectiveness assessment of the AMU reduction plans proposed by the algorithm, a random farm from the dataset was used. This farm had an original AMU of 146.23 TIDDDvet and the mean model prediction out of the 10 iterations was 122.53 TIDDDvet. The measures that were recognised as the farm's strengths were: ‘having a FARM-hygiene lock’, ‘fully disconnecting the drinking water system for cleaning and disinfection’ and ‘storing recognisably the material per stable’. Conversely, ‘having two or more depopulation steps’, ‘not taking specific preventive measures for the material supply’ and ‘not having stable-specific clothing’ were identified as the farm's weaknesses. Seven unique combinations of those weaknesses predicted lower TIDDDvet values compared to the original prediction. Specifically, the biggest TIDDDvet reduction came from changing all weaknesses measures together (Scenario_7: 68.14 TIDDDvet; −44.4% from original prediction). Next, in decreasing order, there was ‘applying one depopulation step’ and ‘take preventive measures for material supply’ (Scenario_6: 73.90 TIDDDvet; −39.7% from original prediction) or ‘have stable-specific clothing available’ (Scenario_4: 87.06 TIDDDvet; −29.0% from original prediction). By applying the one depopulation step alone, a similar goal was achieved (Scenario_5: 86.85 TIDDDvet; −29.1% from original prediction) and with having both specific preventive measures for material supply and stable-specific clothing the prediction was at 94.18 TIDDDvet (Scenario_3: −23.1% from original prediction). Applying the latter two individually also had a reduction effect, but it was marginal. For preventive measures on material supply alone, predicted AMU was 105.6 TIDDDvet and for stable-specific clothing it was 117.01 TIDDDvet (Scenario_2: −13.8% and Scenario_1: −4.5% from original prediction respectively). Scenario_7, which had the biggest reduction (−46.1%) was also the most expensive (€ 8644.87 per year), mainly because of the assumed reduced number of broilers farmed due to the one depopulation step. Excluding that intervention would be equivalent to Scenario_3 (€ 1644.87) and the reduction that could be achieved then was −23.8%. The cost-effectiveness of these two scenarios (Scenario_7: −158.94 €/TIDDDvet; Scenario_3: −58.02 €/TIDDDvet) renders them the ones most worth considering as possible AMU solution plans among the predicted. All results are shown in Table 4.

**Table 4.** Output of the solution plan algorithm for the example farm

Solution plans scenarios	Mean Predicted TIDDDvet	Mean Variance	Original TIDDDvet	Change from Original Prediction	Change from Original Value	Total cost per year	Cost-effectiveness €/TIDDDvet
Original farm	122.53	0.12	146.23	0.0%	−16.2%	€ 0.00	
Scenario_1 i. bqq102_Stable specific clothing is available.^a^	117.01	0.12	146.23	−4.5%	−20.0%	€ 1486.69	−269.14
Scenario_2 i. bqq055_Specific preventive measures are taken for the material supply.	105.60	0.12	146.23	−13.8%	−27.8%	€ 158.18	−9.34
Scenario_3 i. bqq055_Specific preventive measures are taken for the material supply. ii. bqq102_Stable specific clothing is available.	94.18	0.12	146.23	−23.1%	−35.6%	€ 1644.87	−58.02
Scenario_4 i. bqq024_The poultry house is depopulated in 1 step. ii. bqq102_Stable specific clothing is available.	87.06	0.12	146.23	−29.0%	−40.5%	€ 7000.00	−197.32
Scenario_5 i. bqq024_The poultry house is depopulated in 1 step.	86.85	0.12	146.23	−29.1%	−40.6%	€ 8486.69	−237.86
Scenario_6 i. bqq024_The poultry house is depopulated in 1 step. ii. bqq055_Specific preventive measures are taken for the material supply.	73.90	0.12	146.23	−39.7%	−49.5%	€ 7158.18	−147.18
Scenario_7 i. bqq024_The poultry house is depopulated in 1 step. ii. bqq055_Specific preventive measures are taken for the material supply. iii. bqq102_Stable specific clothing is available.	68.14	0.12	146.23	−44.4%	−53.4%	€ 8644.87	−158.94

a Because the number of stables on the farm was unknown, the cost for the stable specific clothing was calculated arbitrarily for two buildings.

## Discussion

In this study, a multi-step analysis was carried out to select the most important biosecurity measures contributing to AMU in broiler farms and to develop a statistical model that, under certain assumptions, can provide indications about which biosecurity measures could be implemented on a given farm for reducing its AMU. The initial step was a risk factor analysis for AMU, followed by the development of an algorithm that assessed and ranked systematically different farm-specific biosecurity intervention scenarios that predicted lower AMU and did not show signs of a non-causative relationship with AMU, including also their associated economic impact. The step of non-causation assessment is of paramount importance here considering the weakness of cross-sectional studies in inferring causality for the observed associations thus some of the variables in the final multivariable Mix-RF model although are good predictors of AMU they are not appropriate solutions for reducing it. Nevertheless, the combination of methods used and developed here can also be applied in more robust study designs toward causal inference increasing thus the reliability of the model and the quality of its propositions even further.

From the final model of the risk factor analysis it was observed that the Gini importance for AMU was higher for the number of broilers per round, the average number of production rounds per year and the number of workers. Based on the literature, farm size and productivity are proxies that define the profile of farms at large and can be associated with both the outcome and the other covariates of interest and thus are usually included in a model as *a priori* control covariates. Studies on pigs and veal calves have shown inconsistent associations between farm size and AMU [40–43]. For poultry, the literature is generally scarce, but in one study from Ghana, farm size was positively associated with AMU and chronic respiratory disease [14]; there though, the farms differ substantially in terms of structure and operations from the farms studied here. In addition, prevalence of early respiratory disease complex has also been seen to be higher in larger broiler farms [44]. Here, we saw that in general the variables in the null model were the most important ones and with regard to farm size, we saw that the bigger and more intensive (i.e. more rounds per year and higher density) the farms were the lower their AMU was, based on RF's effect sizes. By looking at the prototypes though it was seen that, overall, Low Users were farms with a median of 34 550 birds and 7.45 rounds per year compared to 43 000 birds and 6 rounds among High Users. The overall prototypes for the number of birds appeared to be in contrast with that variable's effect size, but these two results originated from two different analyses. The effect size provided an overall estimate of the fixed effect of the predictor on AMU, while the prototypes represented descriptive estimates of that predictor within the heuristically generated Low or High User classes in a given cluster. Discrepancies can therefore occur and could be interpreted as a sign of the unclear true nature of the relationship between the predictor and the response at the overall level (heterogeneous farm profiles) either due to the inability of the current study design to reliably identify causal factors as described above or due to the numerous interactions taking place and thus, looking at the individual clusters is required. For example, within the different clusters, the prototypes of the null model variables showed cluster-specific variation in their associations with AMU. For example, a difference was appreciable between Cluster 1 and Cluster 2 (i.e., 2 out of the 6 distinctive groups identified in terms of common biosecurity practices applied), in which the median numbers of chickens per round for Low – High Users were 18 000– 40 000 and 100 400– 80 000 respectively. Notably, in Cluster 2, 100% of farms were fully disconnecting the water drinking system when cleaning/disinfecting while in Cluster 1 most farms did not and Low Users in Cluster 1 had far more workers than Low Users in Cluster 2 (High Users in both clusters had the same number of workers). Moreover, in Cluster 2, High Users were not using a hygiene lock compared to their Low Users and most farms in Cluster 1. These results illustrate the complex interactions among biosecurity measures and farm characteristics and how they play a crucial role in the ability of farms to operate without depending on antimicrobials. While the importance of those interactions tends to increase with farm size since larger farms are more prone to massive disease spread, at the same time larger farms are also the ones more able (and thus is expected to be more likely) to organise themselves in a more standardised and sometimes efficient way, also in terms of hygiene, thanks to generally greater access to resources, as described earlier [45]. Here this hypothesised correlation was not clearly evident overall and varied across countries. For example the overall biosecurity scores (which can be calculated as described by [15]) only one country showed a medium size correlation with the average number of animals per round (Pearson correlation = 0.46 with *P* value = 0.04).

After the null model variables, next in line (in terms of importance) were the measures related to obliging visitors to check in before entering a farm, having vaccination protocols for non-officially controlled diseases, and performing flock depopulation in two or more steps, which were all associated with higher AMU levels. The unexpected direction of the former two variables could be the result of reverse causality, meaning that the outcome resulted in the exposure and not the other way around, considering that farmers tend to apply those measures when they face a problem and the current study design is cross-sectional, which is prone to this type of bias. Past studies on vaccination programmes in pigs have shown similar effects as well [15, 46]. An extra factor at play here could also be possible shortcomings of current vaccines for food-producing animals with regard to safety, efficacy and/or user-friendliness that limit their efficiency resulting in a not clear-cut relationship with AMU [47, 48]. Regarding flock depopulation steps, [16] provides an informative figure (Fig. 2 in that publication) showing the percentage of flocks being treated altogether (i.e. all 181 farms in the dataset used here) across all production days. There it is seen that the largest treatments occur within the first ten days. The peak in that period was at day three with around 37% of the total flock being treated, reaching then a level lower than 5% at day 13. Subsequently, it increased again to around 12%, but with relatively lower peaks at day 17 and 25, followed by a slowly decreasing trend to 5% till day 30–33. At the usual time of depopulation (i.e., day 30–35), there was a new increase close to 10% and at day 40 it dropped close to 0% due to the waiting times before slaughter. This shows that, indeed, the practice of depopulation may in itself introduce a risk. Nevertheless, the result seen here also includes effects present across the whole production period, such as the increased density of broilers occurring in flock thinning farms before depopulation, which can increase the risk of transmission. Previous research has found conflicting results on whether flock thinning is a risk factor for introduction of infections or not, and it has been suggested that this practice might be confounded by the general hygiene procedures on the farm, or the higher age of the broilers at the time of slaughter for farms with more depopulation steps [17, 49]. Such interactions were also seen here, as for example Cluster 6, which had 100% of Low Users and 95% of High Users applying two or more steps. At the same time, stable-specific clothing and washing hand before entrance was applied mostly by Low Users. In general, preventive measures having an association with lower AMU concurs with various studies on the positive effect of various hygiene practices and barriers on reducing disease prevalence in farms [49–51]. All these factors entail a risk of transmission for the introduction of diseases in the flock [15, 49], but here it is shown that their effect is dependent on the general structure and order of hygienic measures in place. In terms of internal biosecurity, having a farm hygiene lock, having stable-specific clothing, having material recognisably stored per stable, and fully disconnecting and cleaning the drinking water system after every production round, were all associated with lower AMU. The first three factors are in line with previous research on the important role that hygiene protocols and barriers play in reducing AMU [50, 51], while water contamination has been found to be an important source of infection for broilers, specifically for *Campylobacter* [51].

The results showed the high variability in farms' biosecurity practices and that in order to reduce their AMU, interventions needed to be compatible with their profile. The algorithm developed here represents a first step toward providing targeted recommendations for individual broiler farms based on which biosecurity measures will have the greatest effect in terms of AMU reduction. While there is a general relationship between biosecurity and both AMR and AMU [12, 15], most research reports the net effect of risk factors, which makes it difficult to identify which intervention will make the difference in a specific case due to the diverse interaction and confounding effects. However, as described before, several biosecurity measures had relationships with AMU that overall were not in line with the literature or whose associations could not be explained mechanistically. As a result, these features were only used for prediction and were not considered in the AMU reduction plans, given their unclear relationship with the outcome. Finally, the Mix-RF model failed to predict zero AMU values, thereby proposing solutions in cases it was not needed and without being able to assess how to reduce farm's usage to zero. To address this, with enough power it could be possible to treat the response similar to a hurdle model [52] where the risk is parted in two, first the risk of having AMU as a binary response and if so, then to define the extent of the risk. Nevertheless, even in the zero AMU cases, the output of the algorithm would be useful as it shows high risk practices for the farmer and veterinarian to pay attention to.

## Conclusion

In conclusion, several biosecurity-related risk factors for AMU in broiler farms were identified. Using these variables, an algorithm was built that is able to provide quantitative recommendations on which (combinations of) biosecurity measures could be implemented on a given farm in order to expect a reduction in AMU along with the associated costs. This allows individual farms to undertake structural changes in their biosecurity standards based on customised plans that identify the most promising AMU-reducing measures for them. While the method is applicable to different situations and contains several validation steps, further developments are needed to improve our understanding on the interrelatedness of biosecurity measures and how they can influence disease introduction/spread and a farm's need for AMU.

## Data Availability

All data relevant to the study are available from authors at reasonable request and will be anonymised to protect farmers and country privacy. Aggregated AMU data for each country are also provided in the Supplementary material of [21].

## Supplementary material

For supplementary material accompanying this paper visit http://doi.org/10.1017/S0950268822001960. click here to view supplementary material

## Members of the EFFORT consortium

H. Graveland (UUVM), H. Schmitt (UUVM), D. Mevius (WBVR), A. van Essen (WBVR), B. Gonzalez-Zorn (UCM), G. Moyano (UCM), P. Sanders (ANSES), C. Chauvin (ANSES), J. David (ANSES), A. Battisti (IZSLT), A. Caprioli (IZSLT), T. Blaha (TIHO), K. Wadepohl (TIHO), M. Brandt (TIHO), F. Aarestrup (DTU), T. Hald (DTU), S. Duarte (DTU), D. Wasyl (NVRI), D. Krasucka (NVRI), B. Biernacki (NVRI), J. Szumilo (NVRI), H. Daskalov (NDRVI), H. Saatkamp (BEC) and K. Stärk (SAFOSO).
