# Supplementary material for: Biosecurity and antimicrobial use in broiler farms across nine European countries: toward identifying farm-specific options for reducing antimicrobial usage
Source: Epidemiol Infect. 2022 Dec 27;151:e13. doi: 10.1017/S0950268822001960 (PMC9990406; doi:10.1017/S0950268822001960)

**Table S1** Variables from the broiler data set and their descriptive statistics; (Q25: first quartile, Q75: third quartile)

| **Definition of variables** | **N (%)* or Mean (Q25-Q75)** |
| --- | --- |
| ***Y response: Farm antimicrobial use*** |  |
| **TIDDDvet** *normal* | 318.67 (20.96 - 244.2) |
| **TIDDDvet** *transformed by Equation 1* | 4.7 (3.74 - 6.19) |
| ***Null model*** |  |
| bqq002_How many people are working at the poultry farm in total? | 2.86 (1 - 3) |
| bqq010_How many rounds do you have on average? | 6.35 (5.5 – 7.5) |
| bqq011_How many chickens are there for each set-up/round on average? | 62417 (25600 - 83000) |
| ***External biosecurity*** |  |
| bqq023_Are visitors/traders allowed to enter the stables/have direct contact? |  |
| Yes | 61 (33.7%) |
| No | 120 (66.3%) |
| bqq024_In how many steps is the poultry house depopulated? |  |
| One step | 68 (37.6%) |
| Two or more steps | 113 (62.4%) |
| bqq033_When checking water quality, where is the water sample taken? |  |
| At nipple | 108 (60%) |
| Not at nipple | 73 (40%) |
| bqq045_Are visitors obliged to check in before having entrance to the stables? |  |
| Yes | 153 (84.5%) |
| No | 28 (15.5%) |
| bqq050_Are visitors/personnel obliged to wash/disinfect hands before entering? |  |
| Yes | 90 (49.7%) |
| No | 91 (50.3%) |
| bqq055_Are specific preventive measures taken for the material supply? |  |
| Yes | 50 (27.6%) |
| No | 131 (72.4%) |
| bqq060_Is the farm fenced off? |  |
| Yes | 76 (42%) |
| No | 105 (58%) |
| bqq066_Is there natural stagnant water or running water within less than 1km of the farm? |  |
| Yes | 101 (55.8%) |
| No | 80 (44.2%) |
| ***Internal Biosecurity*** |  |
| bqq075_Is there a farm specific protocol for vaccination of non-officially controlled diseases that is complied with? |  |
| Yes | 152 (84%) |
| No | 29 (16%) |
| bqq078_What is the poultry density of the poultry house? |  |
| ≥ 38 kg/m^2^ | 120 (66.3%) |
| ≤ 37 kg/m^2^ | 61 (33.7%) |
| bqq080_Are there disinfection baths for vehicles present at the entry of the company |  |
| Yes | 56 (30.9%) |
| No | 125 (69.1%) |
| bqq084_Is the efficacy of cleaning/disinfection checked by taking a hygienogram? |  |
| Yes | 113 (62.4%) |
| No | 68 (37.6%) |
| bqq087_Is there a FARM-hygiene lock present? |  |
| Yes | 109 (60.2%) |
| No | 72 (39.8%) |
| bqq096_Is the drinking water system fully disconnected and cleaned/disinfected? |  |
| Yes | 65 (35.9%) |
| No | 116 (64.1%) |
| bqq101_Is material stored per stable recognisably? |  |
| Yes | 111 (61.3%) |
| No | 70 (38.7%) |
| bqq102_Is stable specific clothing available? |  |
| Yes | 91 (50.3%) |
| No | 90 (49.7%) |
| ***Housing climate*** |  |
| bqq103b_Is roof ventilation used in the poultry house? |  |
| Yes | 58 (32.0%) |
| No | 123 (68.0%) |
| bqq103e_Is length ventilation used in the poultry house? |  |
| Yes | 61 (33.7%) |
| No | 119 (65.7%) |

*Variables bqq_033, bqq_103b and bqq_103e do not add up to 100% due to missing values. These values were imputed using Multivariate Imputation by Chained Equations (MICE package in R) with Random Forest.

**Figure S1.** Number of farms per country included in each cluster in MDS plot


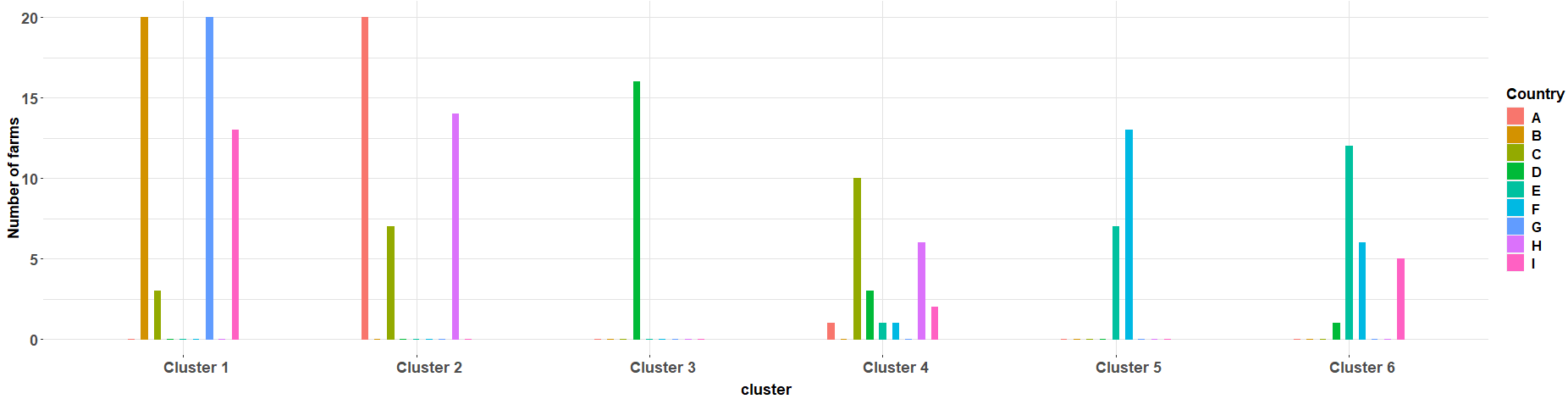

Supplement: Supplementary file 1 [file S0950268822001960sup001.docx]
